# 低氧上调肺腺癌细胞中Annexin A1的表达

**DOI:** 10.3779/j.issn.1009-3419.2012.05.05

**Published:** 2012-05-20

**Authors:** 振红 胡, 瑊 黄, 振华 李, 晓栩 李, 祖红 傅, 海潮 刘, 立彬 官

**Affiliations:** 1 430070 武汉，广州军区武汉总医院呼吸内科 Department of Respiratory, Wuhan General Hospital of Guangzhou Division PLA, Wuhan 430070, China; 2 400038 重庆，中国人民解放军第三军医大学高原军事医学系病理生理与高原生理学教研室 Department of Pathophysiology and High Altitude Medicine, the Third Military Medical University, Chongqing 400038, China

**Keywords:** 肺肿瘤, 低氧, Annexin A1, 活性氧, NF-κB, Lung neoplasms, Hypoxia, Annexin A1, Reactive oxygen species, NF-κB

## Abstract

**背景与目的:**

肿瘤的生长通常会面临缺血缺氧，已有的研究表明膜联蛋白Anx A1（Annexin A1）与肿瘤的关系密切，本研究旨在探讨低氧对肺腺癌细胞Anx A1表达的影响。

**方法:**

将人肺腺癌细胞A549扩增后，分别在常氧（37 ℃、5%CO_2_、21%O_2_）和低氧（37 ℃、5%CO_2_、1%O_2_）条件下培养4 h、12 h、24 h，随后进行RT-PCR，观察Annexin A1 mRNA水平的变化，Western blot方法观察蛋白表达的变化；测定各组细胞中活性氧（reactive oxygen species, ROS）的含量，Western blot检测NF-κB核转位；分别以ROS清除剂N-乙酰半胱氨酸（NAC）和NF-κB抑制剂四氢化吡咯二硫代氨基甲酸脂（PDTC）干预后，测定各组细胞中Anx A1蛋白水平的变化。

**结果:**

RT-PCR结果显示低氧4 h后Anx A1 mRNA水平上升，与常氧组比较有统计学差异（*P* < 0.05），但随后缓慢下降；Western blot结果显示低氧上调A549细胞中Anx A1蛋白的表达，在缺氧4 h时尤为明显；随着细胞缺氧时间的延长，ROS的量也逐渐递增；ROS清除剂NAC和NF-κB抑制剂PDTC明显降低缺氧所致的Anx A1蛋白水平增加。

**结论:**

低氧上调肺腺癌A549细胞中Annexin A1 mRNA和蛋白水平的表达，ROS-NF-κB信号通路可能参与这一过程。

## Abstract (en)

**Background and objective:**

The growth of tumor often faced up with lackness of blood and oxygen, and it has been reported that Annexin A1 may be involved in tumor. The aim of this investigation is to explore the characteristics of expression of Annexin A1 in lung adenocarcinoma A549 cells after hypoxia.

**Methods:**

A549 cells were exposured to either normoxia (21%O_2_) or hypoxia (1%O_2_) condition for 4 h, 12 h, 24 h. The expressions of Annexin A1 mRNA levels were measured by RT-PCR. The expressions of Annexin 1 protein were investigaged by Western blot. The relative content of reactive oxygen species (ROS) were assayed by special kit. The expressions of nuclear translocation of NF-κB was assayed by Western blot; After been treated with ROS scavenger NAC and PDTC, the levels of Annexin 1 protein of A549 cells were measured by Western blot.

**Results:**

Compared with normoxia group, the Annexin A1 mRNA in hypoxia group increased after 4 h, and then decreased gradually; Moreover, Annexin 1 protein levels of A549 cells were also increased when treated with hypoxia. An increaing of ROS production in cells exprosed to hypoxia was detected. NAC and PDTC inhibited hypoxia-induced Annexin A1 increase.

**Conclusion:**

Hypoxia upregulates the expression of Annexin A1 in lung adenocarcinoma A549 cells, in which process ROS-NF-κB may paticipate in.

膜联蛋白Ⅰ（Annexin 1, Anx A1）属于钙离子依赖的磷脂蛋白超家族，具有抗白细胞粘附、诱导分化和调节细胞增殖、凋亡等功能^[1, 2]^。研究^[2, 3]^发现Anx A1在多种肿瘤发生发展过程中的表达发生改变，与肿瘤的分化程度、进展、转移及预后相关，是一种重要的肿瘤标志物。在肿瘤的恶性表现中缺氧起着十分重要的作用，但目前有关缺氧后肺腺癌细胞Anx A1的表达水平变化的报道甚少，因此，本研究观察缺氧后人肺腺癌A549细胞Anx A1表达的变化并探讨其中的可能机制。

## 1 材料与方法

### 1.1 细胞与试剂

人肺腺癌A549细胞株由第三军医大学高原军事医学系提供。Annexin A1抗体（BD公司，美国）；NF-κB抗体（碧云天生物技术研究所）；山羊抗小鼠IgG HRP（北京中杉金桥生物技术有限公司）；RNA提取试剂盒（天根生化科技有限公司），逆转录及PCR试剂（TAKARA，日本）；活性氧（reactive oxygen species, ROS）检测试剂盒（南京凯基生物科技发展有限公司）；N-乙酰半胱氨酸（N-acetylcysteine, NAC）、四氢化吡咯二硫代氨基甲酸脂（pyrrolidine dithiocarbamate, PDTC）（碧云天生物技术研究所）。

### 1.2 细胞分组与处理

用含10%胎牛血清的1640（Gibco，乌拉圭）、100 U/mL青霉素、100 U/mL链霉素配制成1640完全培养基，于35 mm培养皿中培养，每孔1×10^5^个/mL-2×10^5^个/mL，当细胞融合达70%-80%时，取对数生长期细胞进行以下分组实验：①常氧组：37 ℃、5%CO_2_、21%O_2_；缺氧4 h组：37 ℃、5%CO_2_、1%O_2_（下同）；缺氧12 h；缺氧24 h组；②未处理组（C）；缺氧24 h组（H）；NAC组：培养基中加入10 mmol/L的NAC孵育；H+NAC组：先予以10 mmol/L的NAC在37 ℃、5%CO_2_、1%O_2_条件下预处理1 h，再缺氧24 h；PDTC组：培养基中加入30 μmol/L的PDTC孵育；H+PDTC组：先予以30 μmol/L的PDTC在37 ℃、5%CO_2_、1%O_2_条件下预处理10 min，再缺氧24 h。

### 1.3 RT-PCR

取对数生长期的细胞，按试剂盒说明提取总RNA。逆转录条件为37 ℃、15 min，85 ℃、5 s。PCR反应条件为：95 ℃、30 s，95 ℃、5 s，58 ℃、30 s，72 ℃、30 s，72 ℃、5 min，40个循环。引物由英骏公司合成，序列为：Anx A1:上游5'-GCTGTGCATTGTTTCGCTTA-3'，下游5'-GCAGGCCTGGTTTATTGAAA-3'，203 bp。GAPDH：上游5’-GGGAAGGTGAAGGTCGGAGTC-3’，下游5’-CCTGGAAGATGGTGATGGGATT-3’，232 bp。

### 1.4 Wstern blot

用蛋白裂解液裂解细胞后，提取细胞总蛋白（提取细胞核蛋白测定NF-κB的蛋白水平）；BCA法蛋白定量后取30 μg蛋白置12%SDS-PAGE胶中电泳分离；转膜至PVDF膜后置于5%脱脂奶粉中室温封闭1 h；一抗4 ℃孵育过夜；TBST洗涤10 min×3次；二抗1:3, 000稀释后30 ℃孵育1 h，TBST洗涤10 min×3次；ECL化学发光法显色。

### 1.5 细胞ROS含量的测定

处于对数生长期的A549细胞以无血清培养基维持24 h，洗涤换液，对照组在常氧细胞培养箱中培养，缺氧组在37 ℃、5%CO_2_、1%O_2_条件下分别孵育4 h、12 h、24 h后测定各组细胞中ROS的相对含量，按试剂盒操作说明进行ROS的测定。

### 1.6 统计学分析

应用SPSS 17.0统计软件处理，实验所得数据均以Mean±SD表示，两样本均数比较用*t*检验，以*P* < 0.05为差异有统计学意义。

## 2 结果

### 2.1 A549细胞缺氧不同时间后Anx A1 mRNA水平的变化

A549细胞在常氧和缺氧条件下分别培养4 h、12 h、24 h后，采用RT-PCR方法检测Anx A1 mRNA的变化情况。结果显示缺氧4 h时Anx A1 mRNA水平明显升高，与常氧组相比有明显差异（*P* < 0.05）。但随着缺氧时间的延长，Anx A1 mRNA水平逐渐下降，与常氧组相比差异无统计学意义（*P* > 0.05）（图 1）。

**1:**
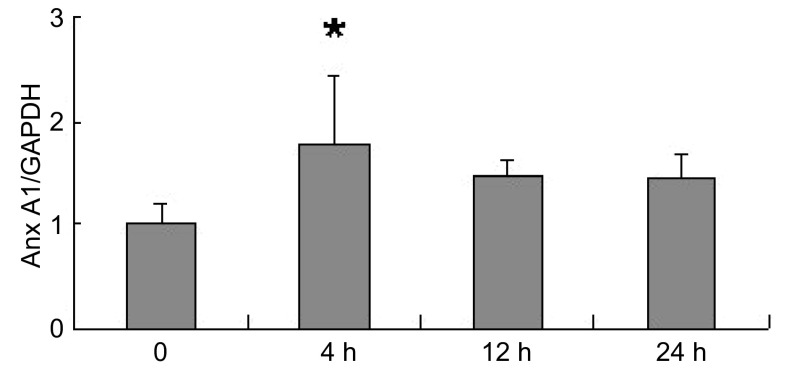
缺氧对Anx A1 mRNA表达水平的影响。^*^：与对照组相比，*P* < 0.05。 The change of Anx A1 mRNA reaction to low O_2_. ^*^: compared with control group, *P* < 0.05.

### 2.2 A549细胞缺氧不同时间后Anx A1蛋白水平的变化

A549细胞在常氧和缺氧条件下分别培养4 h、12 h、24 h，Western blot法检测A549细胞Anx A1的蛋白水平。结果显示缺氧上调Anx A1蛋白的表达，这种上调作用至少持续24 h（图 2）。

**2:**
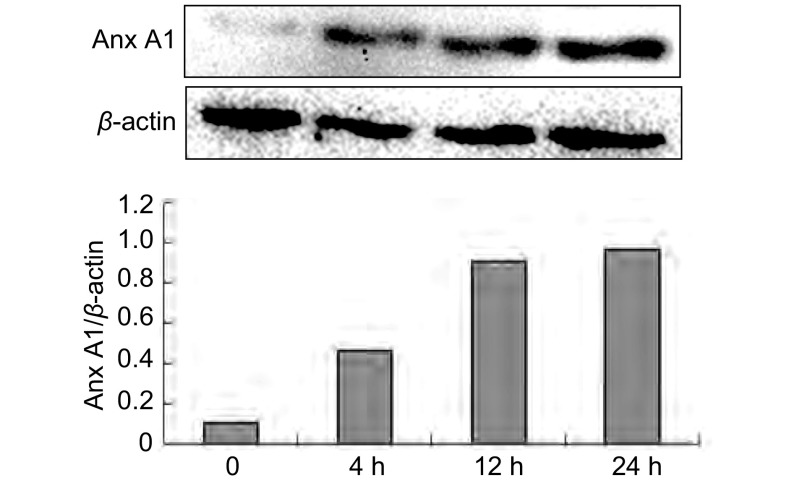
缺氧对Anx A1蛋白水平的影响 The change of Anx A1 protein reaction to low O_2_

### 2.3 缺氧对A549细胞中ROS含量变化的影响

A549细胞ROS的含量在缺氧4 h时即增加，与对照组比较差异有统计学意义（*P* < 0.05）。随后ROS含量继续增加，在缺氧12 h时达峰值，随后缓慢下降，但在24 h内仍高于常氧水平（图 3）。

**3:**
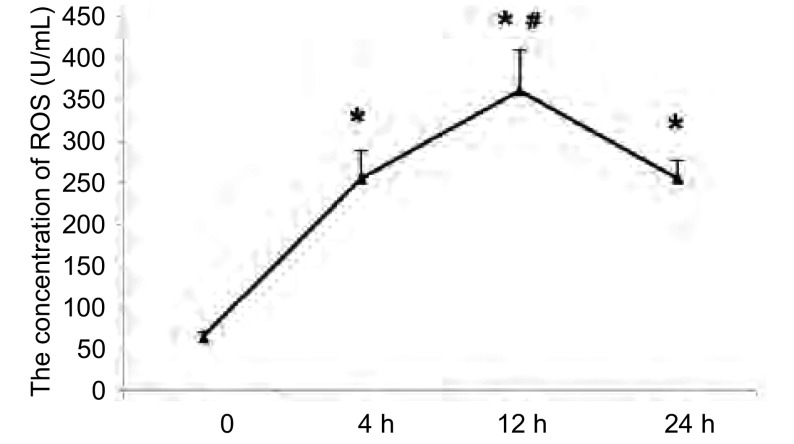
缺氧后A549细胞产生ROS的含量。^*^：与对照组相比，*P* < 0.05；#：与缺血4 h组相比*P* < 0.05。 The level of ROS of A549 reaction to low O_2_. ^*^: Compared with control group, *P* < 0.05; #: Compared with hypoxia 4 h group, *P* < 0.05.

### 2.4 缺氧对NF-κB核转位的影响

我们继续观察缺氧对核转录因子NF-κB核转位的影响。Western blot结果显示：缺氧4 h时NF-κB核转位增加，且在缺氧24 h内，其增加具有时间依赖性（图 4）。

**4:**
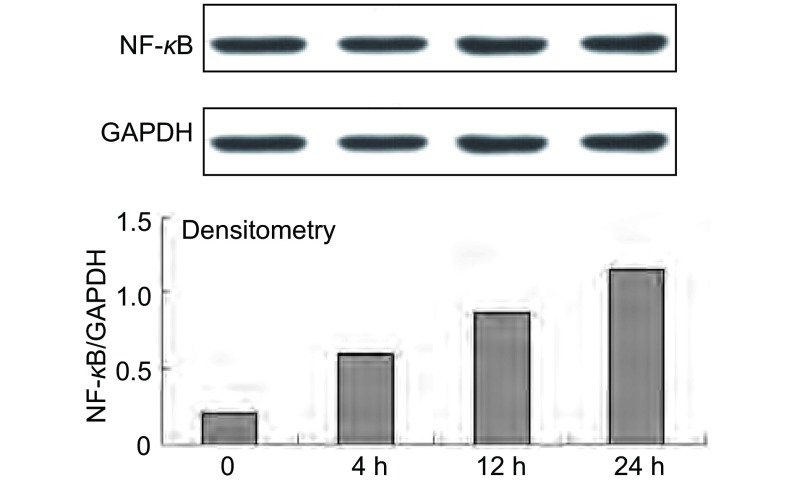
缺氧对NF-*κ*B核转位的影响 The level of NF-*κ*B of A549 reaction to low O_2_

### 2.5 NAC和PDTC对缺氧细胞Anx A1蛋白表达的影响

为进一步研究ROS-NF-κB在缺氧上调过程中的作用，分别用ROS清除剂NAC和NF-κB抑制剂PDTC干预A549细胞。结果发现NAC和PDTC能明显降低缺氧引起的Anx A1蛋白表达增加，但其蛋白水平仍稍高于未处理组（图 5）。

**5:**
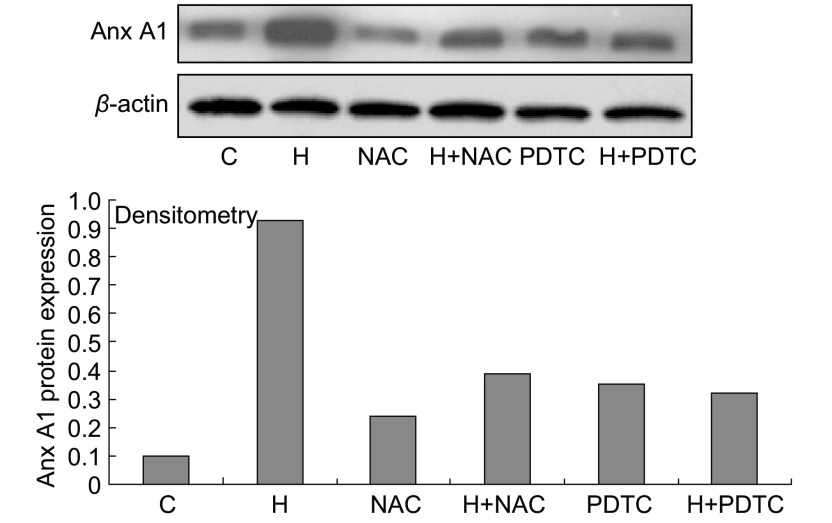
NAC和PDTC对缺氧细胞Anx A1蛋白表达的影响 The change of Anx A1 protein of A549 when treated with NAC and PDTE

## 3 讨论

肿瘤组织生长速度过快常造成肿瘤缺血缺氧，由此产生的相对缺氧环境是肿瘤细胞生长、分化、迁移和浸润的刺激因素^[1]^。Anx A1是一种体内广泛分布的蛋白质，在中性粒细胞、上皮细胞、单核细胞中均高表达^[4]^。我们的研究结果表明，作为一种上皮细胞，肺腺癌A549细胞中Anx A1表达较为丰富。近年来有研究^[5-7]^发现，Anx A1与肿瘤的关系密切，其表达在多种肿瘤的发生发展过程中有明显改变。因此，本研究观察缺氧对肺腺癌A549细胞中Anx A1表达水平的影响，并初步探讨其中可能的信号转导机制。

有研究^[8, 9]^显示，氧自由基水平的增高可导致肿瘤的发生，而肿瘤患者通常也存在机体氧化还原状态的失衡，表明肿瘤与氧自由基之间存在相互联系。本研究结果表明，当A549细胞缺氧后，Anx A1的表达在转录水平和转录后水平均有升高。作为细胞在代谢过程中产生的化学分子，ROS具有广泛的酶激活作用，其可通过损伤DNA、蛋白质并诱导细胞增殖，从而诱导肿瘤的发生发展^[2]^。因此我们推测ROS-NF-κB通路可能参与了缺氧上调Anx A1表达的过程。为了进一步证实这一推测，我们观察到缺氧4 h即能引起细胞内ROS含量明显升高，NAC作为ROS清除剂，能明显降低缺氧引起的Anx A1蛋白表达增加，这提示ROS可能在缺氧上调Anx A1表达的过程中有一定作用。我们通过使用抑制剂后证实，NF-κB可能也参与其中。目前研究已证实ROS可直接激活NF-κB系统，通过磷酸化抑制性蛋白，使其构象发生变化而从NF-κB脱落。NF-κB活化后，核转位调控下游基因的表达^[3]^，以上研究结果均证实了我们的推测。我们还发现，缺氧细胞经清除ROS及抑制NF-κB处理后，Anx A1蛋白的表达仍高于对照组，这说明缺氧诱导的Anx A1表达上调可能还通过其它信号途径（如HIF-1等）来完成。

近年来，Anx A1与肿瘤的关系已成为研究的热点，Anx A1表达水平的改变在肿瘤发生发展中的作用越来越受到关注。Anx A1在不同来源肿瘤的发生过程中发挥着不同的生物学作用及调控机制。缺氧上调肺腺癌细胞中Anx A1表达在肿瘤治疗研究中具有十分重要的意义，但目前关于单纯缺氧对Anx A1表达影响的研究较少，国内有研究表明，肺癌中*Anx A1*基因的mRNA和蛋白水平均上调，且分化程度越低，表达水平越高，提示Anx A1表达水平上调影响肺癌细胞的分化，但具体机制目前尚未完全阐明^[10]^。实体肿瘤内部由于相对缺氧，细胞内氧化还原状态发生改变，通过一系列信号转导途径，影响细胞的增殖、分化、凋亡等。我们的研究初步证实，缺氧不仅能增加肿瘤细胞Anx A1的合成，还能促进Anx A1的释放，Anx A1能介导表达FPR1受体的细胞（如A549细胞）的趋化，其可能参与了肿瘤细胞的分化、增殖，进而影响了肿瘤细胞的浸润转移能力。
